# The spatiotemporal dynamics of longevity-defining cellular processes and its modulation by genetic, dietary, and pharmacological anti-aging interventions

**DOI:** 10.3389/fphys.2012.00419

**Published:** 2012-10-31

**Authors:** Vladimir I. Titorenko, Troy A. A. Harkness

**Affiliations:** ^1^Department of Biology, Concordia UniversityMontreal, QC, Canada; ^2^Department of Anatomy and Cell Biology, University of SaskatchewanSaskatoon, SK, Canada

Aging of multicellular and unicellular eukaryotic organisms is a highly complex biological phenomenon that affects a plethora of processes within cells. This wide array of longevity-defining cellular processes—which are governed by an evolutionarily conserved signaling network—includes oxidative metabolism and protein synthesis in mitochondria, lipid, and carbohydrate metabolism, NAD^+^ homeostasis, amino acid biosynthesis and degradation, ammonium and amino acid uptake, ribosome biogenesis and translation, proteasomal protein degradation, nuclear DNA replication, chromatin assembly and maintenance, actin organization, apoptosis, necrosis, autophagy, protein folding, stress response, signal transduction, cell cycle, and cell growth (Fontana et al., 2010; Kenyon, 2010). The focus of this Frontiers Special Topic Issue is on an important conceptual advance in our understanding of how cells integrate and control these numerous processes and how genetic, dietary, and pharmacological anti-aging interventions extend longevity by altering their functional states and spatiotemporal dynamics. Collectively, the articles in this Issue highlight the various strategies used by evolutionarily diverse organisms for coordinating these longevity-defining cellular processes in space and time, critically evaluates the molecular and cellular mechanisms underlying such coordination, and outlines the most important unanswered questions and directions for future research in this vibrant and rapidly evolving field. Iliadi et al. (2012) eloquently review the use of the fruit fly *Drosophila* as an advantageous model organism to study the mechanisms underlying healthy aging. They provide a broad overview of the important advances that such mechanistic studies in *Drosophila* have made to our understanding of the age-related decline in muscle mass and strength, immune response, stress resistance, sexual behavior, and cognitive function. Walton and Pizzitelli (2012) demonstrate that peroxisome-derived oxidative imbalance in replicatively aging human fibroblasts elicits age-related mitochondrial damage and impairs mitochondrial function. Their findings imply that peroxisomal oxidative damage precedes and is causal to the global mitochondrial dysfunction observed in aging human cells entering a senescent state. Jazwinski and Kriete (2012) explore common principles in the ways by which yeast, nematode, fruit fly, mouse, and cultured human cells respond to partial mitochondrial dysfunction by activating retrograde signaling pathways. The authors critically evaluate how an integration of the retrograde response with other signaling pathways and downstream processes establishes a network that supports cell survival following various external perturbations and under a variety of age-related intracellular stresses. Based on a side-by-side comparison of molecular mechanisms underlying the retrograde responses in yeast and mammalian cells, the authors propose a concept in which these responses operate as a double-edged sword by (1) delaying aging and protecting from external stresses upon their short-term activation; and (2) causing cell death and promoting inflammatory disease if chronically activated in aging. Hou and Taubert (2012) provide excellent insights into important roles for lipid signaling and metabolism in defining longevity of the nematode *Caenorhabditis elegans*. The authors dissect molecular and cellular mechanisms through which the spatiotemporal dynamics of unsaturated fatty acids regulates longevity by modulating the Nuclear Hormone Receptor signaling and maintaining the integrity of various organellar membranes. They also explore the mechanistic links between hydrolysis of neutral lipids and several longevity-defining processes, discuss how lipid-derived signaling molecules impact signaling networks central to longevity assurance, and outline the essential roles of mitochondrial membrane lipids in longevity regulation via multiple cellular pathways. Postnikoff and Harkness (2012) offer us a comprehensive and thought-provoking discussion of how numerous direct and indirect interactions between the Anaphase Promoting Complex and the Forkhead box transcriptional factors orchestrate health- and longevity-related processes in evolutionarily distant organisms by balancing cell-cycle progression and ubiquitin-dependent protein turnover with stress responses and longevity. Their discussion demonstrates that the simple yeast model could be of use to untangle the complex web of Forkhead box protein regulation in higher eukaryotes. Brown and Naidoo (2012) outline recent progress in understanding how intrinsic and extrinsic stresses that impair proteostasis in the endoplasmic reticulum (ER) elicit the unfolded protein response (UPR) signaling pathways that have been shown to play a pivotal role in maintaining cellular protein homeostasis and have been implicated in various age-related diseases. The authors critically evaluate the evidence that progressive age specific changes in the ER stress response and the resulting decline in UPR signaling underlie numerous age-related pathologies, including neurodegenerative diseases, cancer, and inflammation. Kyryakov et al. (2012) provide evidence that the longevity-extending effect of caloric restriction in chronologically aging yeast is due in part to a specific pattern of age-related changes in trehalose concentration elicited by this dietary regimen. They investigate how single-gene-deletion mutations that in chronologically aging yeast alter trehalose concentrations prior to quiescence and following entry into a quiescent state impact lifespan and influence the chronology of oxidative protein carbonylation, intracellular reactive oxygen species, protein aggregation, thermal inactivation of a protein in heat-shocked yeast cells and its subsequent reactivation in yeast shifted to low temperature. Based on their findings, the authors propose a model for molecular mechanisms underlying the essential role of trehalose in defining yeast longevity by modulating protein folding, misfolding, unfolding, refolding, oxidative damage, solubility, and aggregation throughout lifespan. Beach et al. (2012) summarize the evidence that peroxisomes are dynamically integrated into an endomembrane system that governs cellular aging. They discuss various strategies through which peroxisomes are integrated into this endomembrane system, critically evaluate the molecular mechanisms underlying each of these strategies, analyze the age-related dynamics of communications between peroxisomes and other cellular compartments composing the longevity-defining endomembrane system. Communications between peroxisomes and other cellular compartments are explored that influence the development of pro- or anti-aging cellular patterns. Based on the available evidence, the authors propose a model for the integration of peroxisomes into the endomembrane system governing cellular aging. Pallavi et al. (2012) provide insights into molecular and cellular mechanisms underlying longevity-extending and anti-tumor effects of caloric and dietary restriction in mouse models. The authors explore the potential of administering various caloric restriction dietary regimens as a promising way of decelerating the development of breast, colorectal, colon, and ovarian cancers in humans and as a therapeutic approach for cancer treatment in clinical settings.
